# Acid Stress Triggers Resistance to Acetic Acid-Induced Regulated Cell Death through *Hog1* Activation Which Requires *RTG2* in Yeast

**DOI:** 10.1155/2019/4651062

**Published:** 2019-02-25

**Authors:** Nicoletta Guaragnella, Mariarita Stirpe, Domenico Marzulli, Cristina Mazzoni, Sergio Giannattasio

**Affiliations:** ^1^Institute of Biomembranes, Bioenergetics and Molecular Biotechnologies, CNR, Via Amendola 165/a, Bari, Italy; ^2^Department of Biosciences, Biotechnology and Biopharmaceutics, University of Bari “A. Moro”, Bari, Italy; ^3^Pasteur Institute-Cenci Bolognetti Foundation, Department of Biology and Biotechnology ‘Charles Darwin', Sapienza University of Rome, Piazzale Aldo Moro 5, 00185 Rome, Italy

## Abstract

Acid stress causes resistance to acetic acid-induced regulated cell death (AA-RCD) in budding yeast, resulting in catalase activation. In order to explore the molecular determinants of evasion of AA-RCD triggered by acid stress adaptation, we studied the involvement and the possible interplay of the master regulator of transcription high-osmolarity glycerol 1 (*HOG1*) and *RTG2*, a positive regulator of the *RTG*-dependent mitochondrial retrograde signaling. Viability, DNA fragmentation, and ROS accumulation have been analyzed in wild-type and mutant cells lacking *HOG1* and/or *RTG2*. Catalase activity and transcription of *CTT1* and *CTA1*, coding the cytosolic and peroxisomal/mitochondrial catalase, respectively, as well as *Hog1* phosphorylation, were also analyzed. Our results show that *HOG1* is essential for resistance to AA-RCD and its activation results in the upregulation of *CTT1*, but not *CTA1*, transcription during acid stress adaptation. *RTG2* is required for *Hog1*-dependent *CTT1* upregulation upon acid stress, despite failure of *RTG* pathway activation. We give evidence that *Rtg2* has a cytoprotective role and can act as a general cell stress sensor independent of Rtg1/3-dependent transcription.

## 1. Introduction

In multicellular organisms, the controlled suicide of single cells is crucial for development and homeostasis, providing a system that eliminates superfluous cells that might compromise organismal fitness. Similarly, the cellular suicide of a unicellular organism like yeast under certain circumstances provides a system to eliminate cells promoting the survival of the population as a whole. Thus, it is important to understand the mechanisms by which cells activate death or survival pathways in response to environmental changes [1, 2].

Various stress types compromising cell homeostasis elicit the activation of specific adaptive stress response through which extracellular information is converted into rewiring of gene expression aimed at maximizing cell survival [3]. On the other hand, cells of both multicellular and unicellular organisms can succumb through a regulated cell death (RCD) program under extreme conditions [4]. The mechanism by which yeast undergoes RCD in response to acetic acid (AA-RCD) has been investigated in details. Actively dividing yeast cells grown in glucose when shifted to media acidified to pH 3.00 with a strong acid (HCl) and containing 80 mM acetic acid in the undissociated state undergo AA-RCD through a conserved mitochondrial pathway that is characterized by early ROS accumulation, cytochrome *c* release, and mitochondrial dysfunction, as in mammalian intrinsic apoptosis [5]. We have shown that cell incubation at pH 3.00 (acid stress) for at least twenty minutes before adding acetic acid makes yeast adapted to acetic acid stress and fully resistant to AA-RCD [6].

Acid-stressed yeast cells evade AA-RCD due to a specific increase in catalase activity and decrease in ROS accumulation [6, 7]. Moreover, overexpression of *CTT1*, encoding cytosolic catalase T, completely inhibits AA-RCD occurrence [8]. Catalase T expression is upregulated under different stressful conditions, including acid stress [9, 10]. The stress-activated protein kinase (SAPK) high-osmolarity glycerol 1 (*Hog1*) regulates *CTT1* transcription through the transcription factors Msn2/Msn4 [10, 11]. Importantly, *Hog1* has been linked to acetic acid stress adaptation being responsible for the phosphorylation and subsequent degradation of aquaglyceroporin Fps1, required for cellular accumulation of acetic acid at low pH [12, 13].

Yeast mitochondrial retrograde (RTG) signaling is a mitochondria-to-nucleus communication pathway that affects the transcription of nuclear-encoded mitochondrial genes to compensate for mitochondrial dysfunction, thereby restoring metabolic fitness. *Rtg2*, the positive regulator of the RTG signaling, acts as a sensor of mitochondrial dysfunction regulating the nuclear localization of the heterodimeric transcription factor Rtg1/3, which controls the transcription of RTG target genes [14]. RTG pathway activation has been shown to contribute to AA-RCD evasion under glucose derepressing conditions, whereby mitochondrial respiration increased [15]. Interestingly, SAPK *Hog1* has proved to control Rtg1/3 nuclear accumulation and to regulate its binding to chromatin and transcriptional activity in response to osmostress [16].

The aim of this work was to study the role and the possible interplay of HOG and RTG-dependent signaling in AA-RCD evasion of acid-stressed yeast cells. We demonstrated that both *HOG1* and *RTG2* contribute to RCD evasion by protecting cells from oxidative stress and mitochondrial dysfunction in response to acetic acid treatment. The expression of *CTT1*, but not *CTA1*, encoding peroxisomal catalase A, is upregulated by acid stress in an *Rtg2*-dependent manner. Finally, our results indicate that *Hog1* phosphorylation is delayed in the absence of *Rtg2*, indicating for the first time a role of RTG signaling in *Hog1* activation.

## 2. Materials and Methods

We followed the methods of Guaragnella et al. [15].

### 2.1. Yeast Strains, Growth Conditions, and Acetic Acid Treatment

The *S. cerevisiae* strains used in this study were W303-1B (WT) cells (MAT*α ade2 leu2 his3 trp1 ura3*), ∆*rtg2* (*rtg2*∆::*LEU2*), and ∆*hog1* (*hog1*∆::*NAT#2*), which was kindly provided by Prof. Posas, Universitat Pompeu Fabra, Barcelona, Spain. The double mutant ∆*rtg2*∆*hog1* was constructed by replacing *RTG2* with the *LEU2* gene (*rtg2*∆::LEU2 *hog1*∆::NAT#2).

Cells were grown at 30°C in YPD (1% yeast extract, 2% Bacto peptone, and 2% glucose). Acetic acid treatment was carried out as described in [4]. Briefly, cells were grown at 26°C up to exponential phase (OD_600_ = 0.6–0.8) in YPD, resuspended (10^7^ cells/ml) in the same medium adjusted to pH 3.00 with HCl, containing or not (control) 80 mM acetic acid, and incubated for different times at 26°C. Acid-stressed cells were maintained in YPD pH 3.0 medium for 30 minutes before addition of acetic acid. Cell viability was determined by measuring colony-forming units (CFU) after 2 days of growth on YPD plates at 30°C.

### 2.2. TUNEL Assay, Intracellular ROS Detection, and Respiratory Competence Index

DNA fragmentation was detected by TUNEL assay. Acetic acid-treated and control cells (2 × 10^7^) were harvested at 150 min. Briefly, cells were fixed in a 3.7% formaldehyde solution in PBS, digested with 750 *μ*g/ml zymolyase 20T, and incubated in a permeabilization solution (0.1% Triton X-100, 0.1% sodium citrate) for 2 min on ice and then with a 30 *μ*l TUNEL reaction mixture (In Situ Cell Death Detection Kit, Fluorescein, Roche) for 1 hour at 37°C. After incubation, cells were washed, resuspended in PBS, and observed using a Leica TCS SP5 confocal microscope. To detect intracellular H_2_O_2_, 10 *μ*g/ml 2,7-dihydrodichlorofluorescein diacetate (H_2_DCF-DA, Molecular Probes) dissolved in ethanol was added to cells either 30 min before or during cell treatment with or without acetic acid. 2 × 10^7^ acetic acid-treated cells were harvested at 30 min, and oxidation to the fluorophore dichlorofluorescein (DCF) was detected by confocal fluorescence microscopy analysis. Respiratory competence was assessed by measuring cell capacity to grow on nonfermentable and fermentable carbon sources. The same number of cells from acetic acid-treated samples was plated on YPGlycerol (YPG) and YPD, and CFU were counted after 2-3 days. The index of respiratory competence (IRC) was defined as the percentage of cells able to grow in both YPD and YPG [17] and reported as % of acid-stressed WT-adapted cells.

### 2.3. Catalase Activity Assay

Exponentially grown cells (10^8^), directly exposed to acetic acid or pretreated in acidic medium before the exposure, were sedimented by centrifugation, washed once with 50 mM potassium phosphate buffer pH 7.0, and resuspended in 0.3 ml of the same buffer. Cells were broken through mechanical disruption of cell walls with glass beads (0.5 mm BioSpec Products, Bartlesville, OK, USA) by TissueLyser by vortexing eight times for 30 sec with 30 sec intervals in an ice bath and centrifuged for 10 min (15,000×*g*) to clarify the supernatant. A 20–60 *μ*l supernatant was used for enzyme assay. All reagents used for catalytic activity measurements were from Sigma-Aldrich (St. Louis, MO, USA). The total catalase activity was measured spectrophotometrically by monitoring the disappearance of hydrogen peroxide at 240 nm [18]. Protein concentration was determined using the Bio-Rad protein assay (Bio-Rad Laboratories, Hercules, CA), with bovine serum albumin as a standard.

### 2.4. Real-Time Polymerase Chain Reaction (PCR)

The mRNA levels of peroxisomal citrate synthase and peroxisomal and cytosolic catalase-encoding genes (*CIT2*, *CTA1*, and *CTT1*) were determined in exponentially growing cells (OD_600_ = 0.7), after a low pH shift and during acetic acid treatment. 20 ml of cell suspension was centrifuged at 3000×*g*. Cell pellets were either stored at −80°C or immediately used to extract total RNA with Presto Mini RNA Yeast Kit (Geneaid, Taiwan) through mechanical disruption of cell walls with glass beads by TissueLyser (Qiagen). 1 *μ*g RNA (OD_260_/OD_280_ ≥ 1.9) reverse transcription was immediately performed using QuantiTect Reverse Transcription Kit (Qiagen), and cDNA was directly used for real-time PCR analysis or stored at −20°C. Real-time PCR was carried out by QuantiTect SYBR Green PCR Kit (Qiagen) on a QuantStudio 6 Flex instrument from Applied Biosystems using the following primer pairs: for *CIT2*: (F) *5*′-CGGTTATGGTCATGCTGTGCT-*3*′ and (R) *5*′-GGTCCATGGCAAACTTACGCT-*3*′; for *CTA1*: (F) *5*′-CAAGCAAGAAATCTCTACCGCG-*3*′ and (R) *5*′-GCGCTGCTGTATTTGAGGACA-*3*′; for *CTT1*: (F) *5*′-GAGAAAGAGTTCCGGAGCGTGT-*3*′ and (R) *5*′-ATTCTGGTATGGAGCGGCGTA-*3*′; and for *ACT1*: (F) *5*′-CTTTGGCTCCATCTTCCATG-*3*′ and (R) *5*′-CACCAATCCAGACGGAGTACTT-*3*′. The amount of *CIT2*, *CTA1*, and *CTT1* mRNA normalized with *ACT1* mRNA was calculated in arbitrary units (a.u.) using the standard curve method.

### 2.5. Immunoblot Analysis

Samples of total proteins were extracted according to the TCA method previously described [19], separated by electrophoresis on a denaturing gel, and transferred onto a nitrocellulose filter. After the transfer, the membrane was stained with a Ponceau S solution (Sigma-Aldrich) before immunoblotting analysis. Anti-phospho-p38MAP kinase (Thr180/Tyr182) (#9211, Cell Signaling Technology) and *Hog1* (y-215) (sc-9079, Santa Cruz Biotechnology, CA, USA) antibodies (1 : 1000 dilutions) were used to detect phosphorylated *Hog1*p. Immunodetection was performed using enhanced chemiluminescence (ECL; SuperSignal system, Pierce) detected with an X-ray film (Kodak).

### 2.6. Statistical Analysis

Statistical analysis was performed by using paired Student's *t*-test (Excel software).

## 3. Results

### 3.1. AA-RCD Evasion due to Acid Stress Depends on *HOG1* and *RTG2*

**Figure 1:**
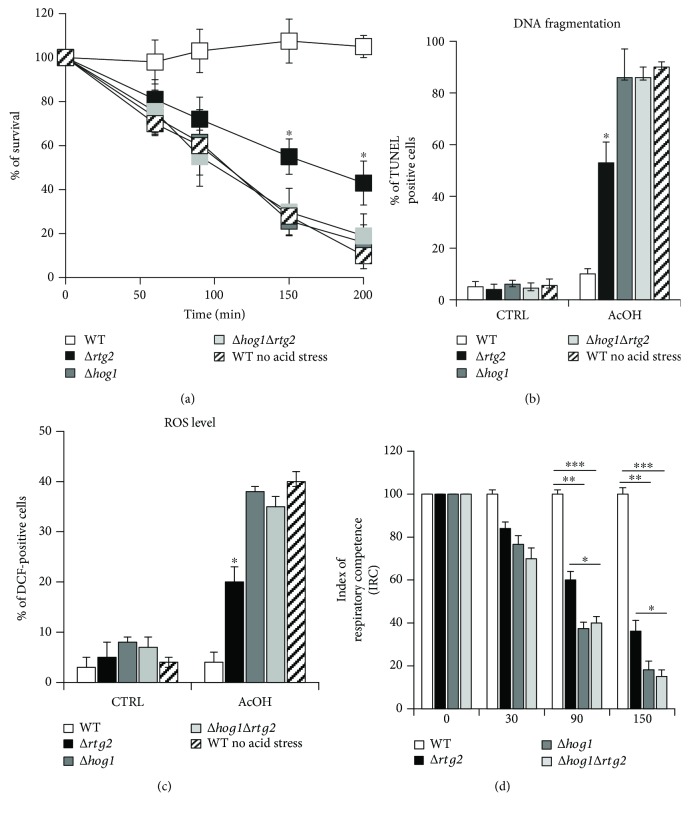
AA-RCD resistance due to acid stress depends on *HOG1* and *RTG2*. (a) Cells were treated with acetic acid (AcOH) after acid stress adaptation, except for WT where indicated. Viability was analyzed at indicated times by measuring colony-forming units (CFU). Cell survival (100%) corresponds to the CFU at time zero. Values are shown as the mean ± standard deviations from four independent experiments. (b, c) DNA fragmentation and intracellular ROS levels were detected by using the TUNEL assay and H_2_DCF-DA probe, respectively. The percentage of TUNEL- and DCF-positive cells is reported at 150 min and 30 min, respectively, with (AcOH) and without (CTRL) acetic acid treatment. In both cases, at least 400 cells were analyzed in three samples from each of the three independent experiments. Paired Student's *t*-test: statistically significantly different with ^∗^*p* < 0.05 when comparing Δ*rtg2* versus WT, with or without acid stress adaptation, Δ*hog1*, and Δ*hog1rtg2*. (d) The index of respiratory competence (IRC) was measured at the indicated times by plating the same cell number on YPD (2% dextrose) and YPG (3% glycerol). The IRC was calculated from three independent experiments as the percentage of cells able to grow in both YPD and YPG and reported as % of acid-stressed WT-adapted cells.

To investigate the role of *HOG1* and RTG pathways in yeast AA-RCD evasion, acid-stressed WT and knockout cells lacking either *HOG1* or *RTG2* or both genes were compared with respect to cell sensitivity to acetic acid. As a control, WT cells were treated with acetic acid without acid stress adaptation. We found that acid-stressed ∆*hog1* cells progressively lose viability which decreased to about 20% at 200 min as for control WT cells that undergo AA-RCD, whereas acid-stressed WT cells remained fully viable, as reported in [6] (Figure 1(a)). Acid-stressed ∆*rtg2* cells showed 50% viability after 200 min whereas ∆*hog1*∆*rtg2* behaved similarly to ∆*hog1* cells (Figure 1(a)). The specific death rates of acid-stressed ∆*hog1* and ∆*hog1*∆*rtg2* cells (0.015 min^−1^) were similar to the ones measured in WT cells undergoing AA-RCD.

In order to assess the nature of cell death, DNA fragmentation was analyzed in acid-stressed cells, both with and without (control) acetic acid treatment. About 90% of ∆*hog1* and ∆*hog1*∆*rtg2* cells treated with acetic acid were positive to TUNEL assay at 150 min (Figure 1(b)). This percentage was similar to the one measured in dying WT cells. Differently, less than 20% of acid-stressed WT cells showed DNA fragmentation. In ∆*rtg2* cells, the percentage of DNA fragmentation was more than 50% at 150 min (Figure 1(b)). In the absence of acetic acid treatment, less than 5% of cells were TUNEL positive in all cell types.

Since early ROS accumulation is a typical marker of yeast AA-RCD [5], intracellular hydrogen peroxide levels were measured in WT and mutant cells at 30 min. In ∆*hog1* and ∆*hog1*∆*rtg2* cells, the percentage of DCF-positive cells increased from less than 10% in the control to about 40% following acetic acid treatment (Figure 1(c)). Less than 5% acid-stressed WT cells, which evade AA-RCD, accumulated hydrogen peroxide. Interestingly, in ∆*rtg2* cells, ROS accumulation was significantly higher than in acid-stressed WT-adapted cells (20% of positivity to DCF versus 4% measured in WT cells) but still lower than ∆*hog1* and ∆*hog1*∆*rtg2* (Figure 1(c)).

Mitochondrial dysfunction is another hallmark of AA-RCD [20]. Thus, to assess whether the loss of mitochondrial function occurred in the death-sensitive mutants, the index of respiratory competence (IRC) was evaluated as a function of time in acid-stressed ∆*hog1*, ∆*rtg2*, and ∆*hog1*∆*rtg2* cells treated with acetic acid. A reduction of IRC was observed in all cases, with a more drastic decline in functionality for ∆*hog1* and ∆*hog1*∆*rtg2* mutants with respect to ∆*rtg2* cells, about 80% versus 60%, respectively, compared to WT-adapted cells after 150 min (Figure 1(d)).

Overall, these results show that *HOG1* and partially *RTG* signaling contribute to AA-RCD evasion due to acid stress.

### 3.2. *RTG2* Is Required for the Full Activation of Catalase T in Acid-Stressed Cells Resistant to AA-RCD

**Figure 2:**
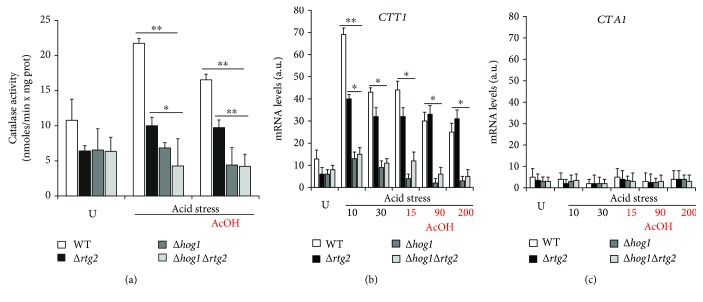
*CTT1* mRNA upregulation triggered by acid stress is impaired in ∆*rtg2* mutants. Cells were grown up to exponential phase (U), incubated at low pH (acid stress), and treated with acetic acid (AcOH) at the indicated times, respectively. (a) Catalase activities were determined on cell extracts at 30 min of acid stress and after 30 min of acetic acid exposure as described in Materials and Methods. Values are shown as the mean ± standard deviations from three independent experiments. Paired Student's *t*-test: statistically significantly different with ^∗∗^*p* < 0.005 when comparing WT versus Δ*rtg2*, Δ*hog1*, and Δ*hog1rtg2* and comparing Δ*rtg2* versus Δ*hog1* and Δ*hog1rtg2* at low pH and with acetic acid and with ^∗^*p* < 0.05 when comparing Δ*rtg2* versus Δ*hog1* and Δ*hog1rtg2* at low pH. (b, c) *CTT1* and *CTA1* mRNA levels were measured by real-time PCR at the indicated times in WT and mutant cells. In both cases, mRNA levels were normalized to that of *ACT1* mRNA and reported as means with standard deviation in fluorescence arbitrary units (a.u.). Paired Student's *t*-test: statistically significantly different with ^∗∗^*p* < 0.005 when comparing WT versus Δ*rtg2*, Δ*hog1*, and Δ*hog1rtg2*; with ^∗^*p* < 0.05 when comparing Δ*rtg2* versus Δ*hog1* and Δ*hog1rtg2* at 10 min low pH; and with ^∗^*p* < 0.05 when comparing WT and Δ*rtg2* versus Δ*hog1* and Δ*hog1rtg2* at 30 min low pH and with acetic acid.

Catalase activity, almost undetectable in exponentially growing WT cells, is specifically triggered by extracellular acid stress reaching its maximum after 30 min and remaining high in cells protected from AA-RCD. Thus, it represents a hallmark of cells evading AA-RCD [6]. Since *Hog1* is involved in the osmotic regulation of *CTT1* transcription [10], catalase activity was measured in WT and knockout cells either in normal growth conditions without acetic acid (untreated) or at 30 min of acid stress before and after acetic acid addition (Figure 2(a)). As expected, catalase activity was high in WT cells at low pH and remained higher than in untreated cells after the addition of acetic acid. All mutant cells exhibited an overall significant reduction of catalase activity. However, although catalase activity showed a slight but significant increase in acid-stressed Δ*rtg2* cells with and without acetic acid, it remained virtually constant in ∆*hog1* and ∆*hog1*∆*rtg2* cells in all conditions. These data suggest that *RTG2* participates in the *HOG1*-dependent catalase activity increase caused by acid stress. This is in agreement with the partial and full sensitivity to AA-RCD of *rtg2* and *hog1* mutants, respectively.

**Figure 3:**
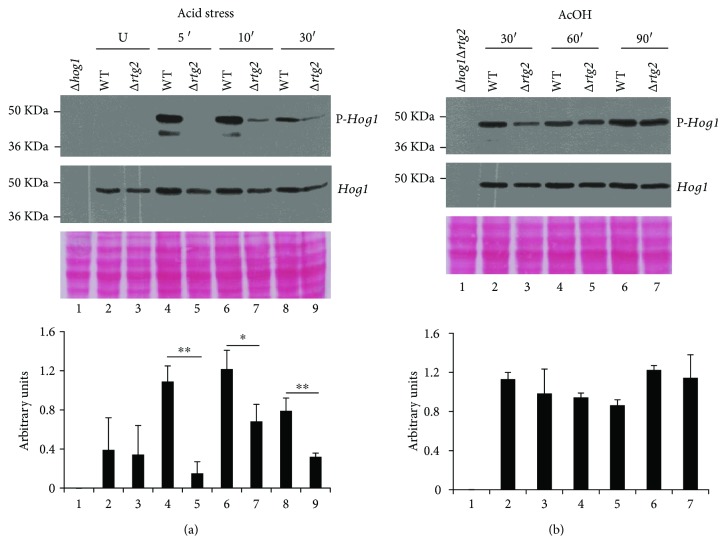
*Hog1* phosphorylation in WT and ∆*rtg2* mutants. (a) Cells were grown up to exponential phase (U), incubated at low pH, and (b) treated with acetic acid (AcOH). Cell protein extracts were prepared from WT and ∆*rtg2* cells at the indicated times and analyzed by immunoblot with anti-P-*Hog1*p and anti-*Hog1*p antibodies. Cell extracts from ∆*hog1* and ∆*hog1*∆*rtg2* cells have been analyzed as a negative control. Ponceau staining was used as loading control. The histogram reports the ratio between phosphorylated (P-*Hog1*) and total *Hog1* as quantified by densitometric analysis of the film normalized with Ponceau staining and expressed in arbitrary units. Reported values are the means ± standard deviations of three independent experiments. Paired Student's *t*-test: statistically significantly different with ^∗∗^*p* < 0.05 and ^∗^*p* < 0.1 when comparing Δ*rtg2* versus WT under acid stress.

The impairment of the *RTG* pathway and/or *HOG1* deletion partially or fully restored, respectively, AA-RCD in acid-stressed cells with concomitant early accumulation of ROS and a decrease in catalase activity (see Figures 1(c) and 3). Since *Hog1* controls the regulation of *CTT1* transcription through the transcription factors Msn2/4 [21], we wondered whether *RTG2* might have a role in the expression of either *CTT1* or *CTA1*, coding the cytosolic and peroxisomal catalase, respectively. To this aim, *CTT1* and *CTA1* mRNA levels were measured in WT, ∆*rtg2*, and/or ∆*hog1* cells either in untreated cells or acid-stressed cells before and after acetic acid addition. We found that the *CTT1* transcription profile mirrored catalase activity measured in the same cells and under the same conditions (Figure 3(b)). Indeed, *RTG2* deletion strongly inhibited *Hog1*-dependenttranscription in untreated and acid stress conditions. The *CTT1* mRNA level is rapidly and transiently increased up to 5-fold after 10 minutes of acid stress (about 70 a.u.) and decreased to about 45 a.u. after 30 min in WT cells. No changes were observed for *CTT1* transcription following acetic acid treatment. On the other hand, *CTT1* expression was 40 a.u. after 10 min of acid stress and remained constant also during the acetic acid treatment in ∆*rtg2* cells (Figure 2(b)). It is of note that in all conditions tested, no *CTA1* transcription was observed in any strains analyzed (Figure 2(c)).

These data showed that only catalase T activity is responsible for AA-RCD evasion in acid-stressed cells. They also confirm that *CTT1* mRNA upregulation following acid stress strictly depends on SAPK *Hog1* kinase and gave the first evidence that *Rtg2* is required for *Hog1*-dependent *CTT1* mRNA upregulation in response to acid stress.

### 3.3. *RTG2* Deletion Causes a Delay in Hog1 Phosphorylation

**Figure 4:**
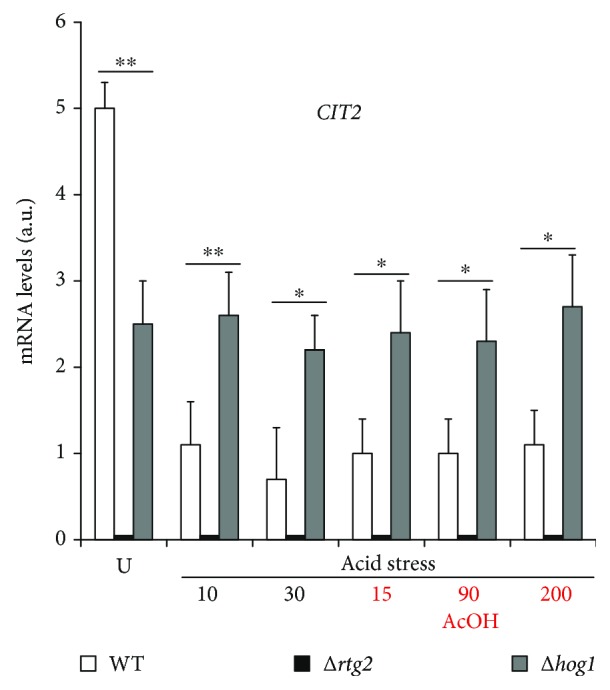
*CIT2* expression in ∆*hog1* mutants. Cells were grown up to exponential phase (U), incubated at low pH, and treated with acetic acid (AcOH). *CIT2* mRNA levels were measured by real-time PCR at the indicated times in WT and mutant cells from three independent experiments. *CIT2* mRNA levels were normalized to that of *ACT1* mRNA and reported as means with standard deviation in fluorescence arbitrary units (a.u.). Paired Student's *t*-test: statistically significantly different with ^∗∗^*p* < 0.1 when comparing WT versus Δ*rtg2* and Δ*hog1* in untreated cells and at 10 min low pH and with ^∗^*p* < 0.5 when comparing WT versus Δ*hog1* and Δ*rtg2* at 30 min low pH and with acetic acid.

*Hog1* activation occurs through its phosphorylation and translocation from the cytoplasm to the nucleus, which is required for transcriptional regulation [22]. Thus, to gain insights into how *RTG2* may alter *Hog1*-dependent transcription, we monitored the *Hog1* phosphorylation state in acid-stressed WT or *RTG2*-lacking cells with or without acetic acid treatment (Figure 4). Under unstressed conditions, the phosphorylated protein was almost undetectable in both WT and mutant cells. *Hog1* phosphorylation ensued in acid-stressed WT, but not in ∆*rtg2* cells after 5 and 10 minutes. At 30 minutes, while phosphorylation increased in ∆*rtg2* cells, a reduction in the amount of phosphorylated protein was observed in WT cells (Figure 3(a)). During acetic acid treatment, *Hog1* showed a level of phosphorylation in WT cells virtually similar to that of ∆*rtg2* cells at 30, 60, and 90 min (Figure 3(b)).

Thus, *RTG2* can modulate the phosphorylation of *Hog1* causing a delay in the activation of *Hog1* kinase at least in response to acid stress.

### 3.4. Rtg2 Does Not Activate Rtg1/3-Dependent Retrograde Target Gene Transcription in Acid Stressed Cells

**Figure 5:**
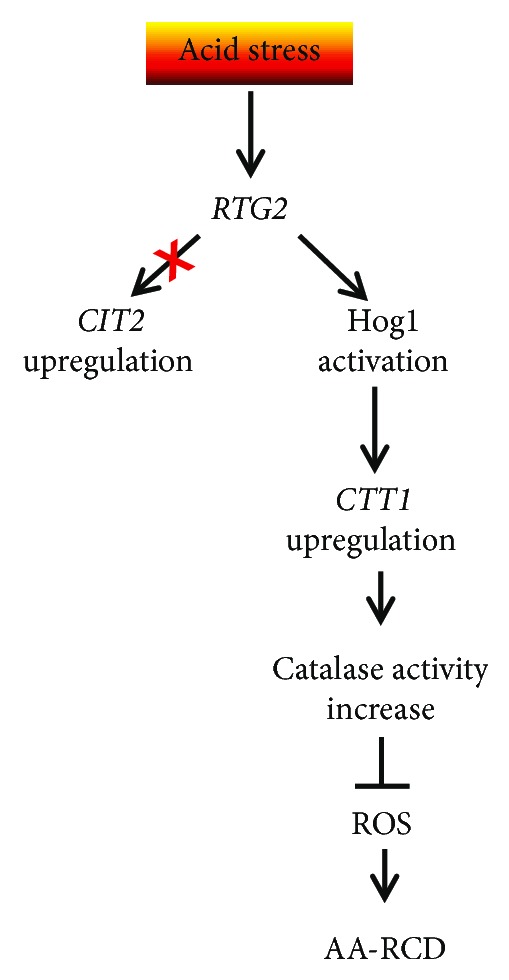
A model for the AA-RCD adaptive pathway due to acid stress. Acid stress triggers resistance to AA-RCD through a pathway in which *RTG2* does not activate the *CIT2* upregulation, i.e., mitochondrial retrograde response, but it is required for *Hog1* activation which controls the transcriptional upregulation of *CTT1*, encoding cytosolic catalase. The concomitant increase of catalase activity inhibits the ROS-dependent pathway causing AA-RCD.

We have previously shown that yeast cells grown in raffinose, which differently from glucose, promotes respiration, evade AA-RCD due to RTG pathway activation [15, 23]. In order to check if RTG pathway activation can contribute to AA-RCD evasion of acid-stressed cells, the mRNA level of *CIT2*, encoding the peroxisomal isoform of citrate synthase, whose upregulation is a hallmark of Rtg1/3-dependent transcription activation [14], was measured in WT and ∆*hog1* mutant cells either in normal growth conditions without acetic acid (untreated) or during acid stress before and after acetic acid addition (Figure 4). The level of expression was about 5 a.u. in the absence of stress. Interestingly, about a 2-fold reduction of the *CIT2* mRNA level of expression was measured in ∆*hog1* cells under normal growth conditions. The *CIT2* mRNA level showed about an 80% decrease in acid-stressed WT cells with respect to normal growth conditions and exhibited a virtually constant value either in the absence or in the presence of acetic acid. This shows that the RTG pathway was not activated by acid stress. On the other hand, the *CIT2* mRNA level remained unchanged as compared with normal growth conditions in acid-stressed ∆*hog1* cells with or without acetic acid treatment (Figure 5). As expected, *CIT2* expression was completely abolished in the ∆*rtg2* mutant.

These data demonstrate that the RTG signaling does not contribute to AA-RCD evasion in cells adapted by acid stress. In addition, *Hog1* is required for the full transcriptional activation of *CIT2* in normal growth conditions as well as for the *CIT2* transcription regulation in response to stress.

## 4. Discussion

Yeast cells can evade AA-RCD either upon acid stress adaptation, due to the cytoprotective role of the catalase upregulation, or under conditions of concomitant derepression of mitochondrial respiration and RTG pathway activation, like in raffinose-grown cells [6, 8, 15]. In this work, we showed a mutual interplay between *RTG2*, a positive regulator of the mitochondrial RTG pathway, and SAPK *HOG1*, the master regulator of catalase gene transcription, in coordinating stress response and modulating resistance to AA-RCD due to acid stress adaptation.

Our data demonstrate that both *HOG1* and *RTG2* contribute to AA-RCD evasion, with an essential requirement of *HOG1* (Figures 1(a) and 2(b)). These two genes seem to exert their protective role against acetic acid toxicity by increasing oxidative stress resistance and preserving mitochondrial function (Figures 1(c) and 1(d)). The requirement of *RTG2* for *CTT1* activation, as shown in Figure 3(a), supports the involvement of the RTG pathway in oxidative stress resistance as already shown in [13, 24, 25]. Complete preservation of mitochondrial function in acid stress cells evading AA-RCD was abolished by *HOG1* deletion, confirming the link of the MAPK pathway with mitochondrial dysfunction already observed upon osmotic stress and in pathogenic fungi [24, 26, 27].

Here, we showed that specific activation of cytosolic catalase T, but not peroxisomal/mitochondrial catalase A, is responsible for acid stress cell evasion of AA-RCD (Figure 2). This is in agreement with previous findings that *CTT1* overexpression can fully prevent AA-RCD [8]. Thus, detoxification of hydrogen peroxide together with catalase activation appears to be a key condition for acetic acid tolerance and in general stress cotolerance as reported in [28].

How *RTG2* interacts with *HOG1* to regulate *CTT1* transcription remains to be established. Our analysis of *Hog1* phosphorylation *en route* to acid stress clearly shows that *RTG2* deletion significantly delays *Hog1* activation through phosphorylation, suggesting possible interactions with *Hog1*-specific kinases (Figure 3). On the other hand, *HOG1* has been involved in the regulation of Rtg1/3 complex activity in response to osmostress [16]. According to these results, our data showed that *HOG1* could exert a modulatory effect on Rtg1/3-dependent transcription depending on the metabolic state of the cell: *Hog1* is required for the full transcriptional activation of the *CIT2* gene in normally growing cells, but it negatively regulates RTG pathway activation in stressed cells (Figure 4). This is in agreement with the increase of *CIT2* expression occurring in a *Hog1*-dependent manner upon osmostress [16]. Overall, our data clearly demonstrate the interplay between *Rtg2* and *Hog1* (Figure 5), whose mechanism deserves further investigations. However, since *Rtg2* can bind to chromatin at the promoters of regulated genes as a component of a SAGA-like complex, named SLIK [29], and an interaction between *Hog1* SAPK and SAGA complex has been previously reported upon severe osmostress [22, 30–32], we can hypothesize that *Rtg2* and *Hog1* interact at the level of epigenetic regulation of gene expression. In any case, our results clearly show a strict cooperation of HOG and RTG pathways to induce a hormetic response of yeast cells to different kinds of stresses.

It is worth noting that HOG pathway activation can rescue cells from various cellular dysfunctions caused by the impaired biosynthesis of complex sphingolipids through a yet unknown molecular mechanism [33]. It has been suggested that the HOG pathway could sense impaired formation of lipid microdomains, which are formed by complex sphingolipids and sterol molecules [34]. Since others and we have shown that ceramide production contributes to mitochondrial dysfunction and AA-RCD [35, 36], our results support the hypothesis (it is tempting to speculate) that acid stress could activate *Hog1* through a mechanism alike that ensues a sphingolipid metabolism defect.

Differently from raffinose-grown cells which evade AA-RCD mostly due to RTG pathway activation [15], acid stress does not activate Rtg1/3-dependent mitochondrial retrograde target gene transcription for cell adaptation (Figure 4). Thus, different branches of the *RTG*-dependent signaling can be activated depending on the type of external stimuli, suggesting that *RTG2* may have a complex role in the integration of different stress signaling pathways through yet unknown regulatory elements [37].

## 5. Conclusions

By further exploring the molecular basis of yeast tolerance to acetic acid, this work highlights the importance of detailed understanding of the interplay between signaling pathways in stress response. In particular, it provides novel insights into the strict relationship between cell stress adaptation and oxidative stress resistance ensuing from the cooperation of *RTG*- and *HOG1*-mediated stress signaling pathways. When activated, these pathways converge to produce a hormetic response in cells undergoing environmental stresses. The discovery of the new role for *RTG2* in modulating *Hog1* activation will deserve deeper investigations to understand conditions and modes of this interaction upon stress. These results could be relevant for certain industrial microbial applications in which acetic acid accumulation is detrimental and the improvement of strain resistance to acetic acid is highly desirable.

## Data Availability

The data used to support the findings of this study are available from the corresponding author upon request.
